# Management of Dry Eye Disease for Intraocular Lens Power Calculation in Cataract Surgery: A Systematic Review

**DOI:** 10.3390/bioengineering11060597

**Published:** 2024-06-11

**Authors:** Atsushi Kawahara

**Affiliations:** Yoshida Eye Hospital, 2-31-8, Hondori, Hakodate 041-0851, Hokkaido, Japan; atsusi-k@coral.plala.or.jp; Tel.: +81-138-53-8311

**Keywords:** dry eye disease (DED), dry eye, cataract surgery, intraocular lens (IOL), power calculation, keratometry, refractive error, 2nd International Dry Eye Workshop of the Tear Film and Ocular Surface Society (TFOS DEWS II), tear film break-up time (TBUT), diquafosol

## Abstract

Cataracts are characterized by the crystalline lens of the eye becoming cloudy, and dry eye disease (DED) is a multifactorial disease in which the homeostasis of the tear film is lost. As the prevalence of both diseases increases with age, there is a high prevalence of DED among patients who are candidates for cataract surgery. In recent years, cataract surgery has evolved from vision restoration surgery to refractive surgery. To achieve good surgical outcomes, it is necessary to minimize postoperative refractive error in intraocular lens (IOL) power calculation, which requires accurate preoperative keratometry measurements. A stable tear film is important for the accuracy and reproducibility of keratometry measurements, and DED may have a deleterious effect. In this study, original articles that focused primarily on findings related to this topic were evaluated. A systematic review was performed according to the Preferred Reporting Items for Systematic Reviews and Meta-Analyses (PRISMA) guidelines. Although appropriate DED diagnoses were not presented in the articles evaluated in this review, it was confirmed that the clinical signs of DED, particularly the shortening of the tear film break-up time (TBUT), negatively impact IOL power calculations. Improvement in these clinical signs might mitigate the negative effects on these calculations.

## 1. Introduction

Cataracts are characterized by opacity of the lens of the eye, with the most common form being age-related cataracts. The disease is more common in women and its prevalence increases with age, with onset around age 50 [1]. Survey statistics for 2020 revealed that approximately 78 million adults over the age of 50 worldwide were visually impaired due to cataracts, representing a 175% increase in patients in the 30 years leading up to that year [2]. Therefore, the demand for cataract surgery has increased in recent years, leading to advances in medical devices, surgical techniques, and intraocular lenses (IOLs), and resulting in the evolution of cataract surgery from vision-restoring surgery to refractive surgery [3]. Good surgical outcomes require accurate keratometry, axial length and anterior chamber depth measurements, and the use of IOL power formulas with minimal postoperative refractive error [4]. Accurate preoperative keratometry measurements via biometry are especially important, as an error of 1 diopter (D) in the keratometry measurement will result in an error of approximately 1 D in the IOL power calculation [5].

Dry eye disease (DED) is a multifactorial disorder characterized by a loss of tear film homeostasis [6]. The number of patients with DED is increasing worldwide and the disease is more common among Asian ethnic groups and women; the prevalence also increases with age [7,8]. Therefore, patients indicated for cataract surgery have a higher prevalence of DED than the general population [9]. The stability of the tear film is mainly indicated by the tear film break-up time (TBUT), and in recent years the number of patients with short TBUT-type DED has increased [10]. In one study [11], preoperative screening tests for cataract surgery showed a shortened TBUT in approximately 60% of patients. The tear film is the first refractive surface in the ocular optical system, and it is optically important to maintain the constancy of its smoothness and regularity [12]. This underscores the importance of the stability of the tear film for the accuracy and reproducibility of keratometry measurements, which depend on the reflection of tear fluid on the corneal surface [13]. Therefore, cataract surgeons must always consider the preoperative diagnosis and effective management of DED [14,15].

DED was first defined by the National Eye Institute/Industry Workshop in 1995 [16]. This definition suggests that decreased tear fluid volume is central to the characteristics of DED. The Delphi consensus group then revised the definition in 2006, including subjective symptoms in the diagnostic criteria and proposing the name dysfunctional tear syndrome for DED [17]. In 2007, the International Dry Eye Workshop (DEWS), under the auspices of the Tear Film and Ocular Surface Society (TFOS), further revised the definition and diagnostic criteria, clearly stating that DED is a multifactorial disease, with subjective symptoms, such as decreased tear fluid volume and ocular surface damage, essential diagnostic criteria [18]. However, it was noted that further revision of the definition and diagnostic criteria was necessary because of the high prevalence of short TBUT-type DED in practice. Thus, new definitions and diagnostic criteria were developed to address this and other concerns, and these were presented in the 2017 report of the 2nd International Dry Eye Study Group (TFOS DEWS II). The TFOS DEWS II proposed that the characteristic feature of DED is the loss of homeostasis of the tear film. This TFOS DEWS II Report is now widely used as the global standard for DED in clinical practice and research.

In recent years, an increasing number of review articles have focused on the impact of DED on the perioperative management of patients undergoing cataract surgery. However, most articles discuss the preoperative, intraoperative, and postoperative impact separately, with little discussion of the impact on IOL power calculation. Some articles review the topic but, to my knowledge, none of them adequately discuss DED, the focus of this review, using the TFOS DEWS II Report as a basis. The purpose of this paper is to first evaluate DED diagnosis and management in the original articles in terms of the TFOS DEWS II Report, which has been applied in many studies, and then to discuss its impact on IOL power calculations. In addition, based on these considerations, preoperative management strategies for IOL power calculations in cataract patients with DED are proposed.

## 2. Materials and Methods

This systematic review was conducted in accordance with the Preferred Reporting Items for Systematic Reviews and Meta-Analyses (PRISMA) guidelines. The protocol for the systematic review was registered with the International Prospective Register of Systematic Reviews (PROSPERO) and assigned the unique identifier CRD42024544532. The population–intervention–comparison–outcome (PICO) style for this review was “in patients scheduled for cataract surgery with concomitant DED (P), does DED treatment and/or biometry measurement (I) intervention improve postoperative refractive error (O) compared to subjects without intervention or with no concomitant DED (C)?”

A literature search of the PubMed electronic bibliographic database was conducted on 9 May 2024. The search terms were “Cataract surgery” AND “Dry eye”. Next, before screening, original articles published in the last 10 years were extracted. The 10-year period was chosen because of recent developments in cataract surgery practice (examination equipment, surgical instruments, and techniques) and because of recent changes and advances in DED practice, as described in the Introduction. Inclusion criteria were as follows: adult human participants scheduled for cataract surgery and diagnosed with DED, participants with preoperative DED treatment and/or biometry measurement intervention, preoperative biometry and pre- and postoperative refractive outcome measures, original articles published in English, a sample size of at least 20 participants per group, randomized or non-randomized controlled trials, observational studies, and case series. Exclusion criteria included papers that were review articles or case reports.

The author (AK) screened the titles and abstracts to assess their relevance and consistency with the objectives of this systematic review. This initial screening was followed by a full-text review, which extracted information from each selected study, including participant characteristics and conclusions drawn. Only studies with appropriate methodologies and results were then included in the systematic review based on PICO, and the above inclusion and exclusion criteria, and these were systematically evaluated to ensure that the study results were valid and reliable. With respect to the data integration process, the first step was a thorough extraction of relevant data from each study, including participant characteristics, number of participants, intervention details, and outcome measures. Subsequently, a qualitative analysis of outcomes, including an analysis of risk of bias using the risk-of-bias visualization tool [19], was performed. The process of searching, selecting, evaluating, extracting, and analyzing the results was reviewed by the review team (AK and SY).

## 3. Results

**Figure 1:**
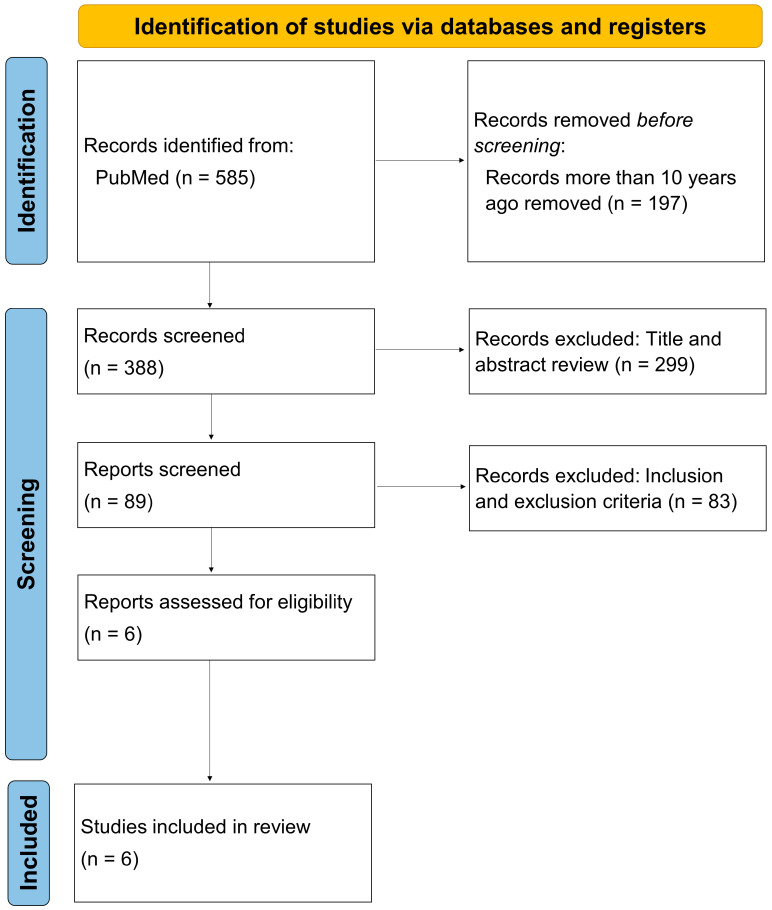
PRISMA flowchart.

**Figure 2:**
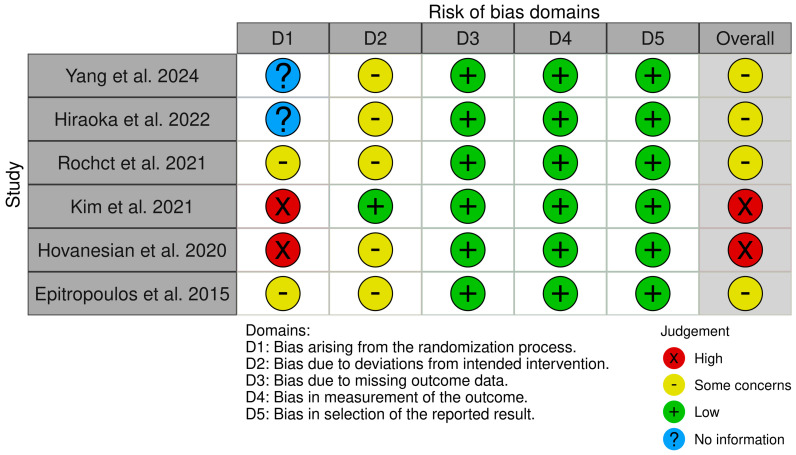
Assessment of the risk of bias in the studies included using the risk-of-bias visualization tool [13,20,21,22,23,24].

The PRISMA flowchart is shown in Figure 1. The initial electronic search identified 585 references. Then, after review according to the inclusion, exclusion, and other criteria, a total of six original articles [13,20,21,22,23,24] were included in this review. A brief summary of the papers is presented in Table 1 and Table 2. With regard to the analysis of risk of bias, two studies were classified as high risk for bias and four as moderate risk. No studies were classified as low risk. The results were generally due to the inability to blind participants and the responsible physicians because of the nature of the intervention applied, and the non-randomized nature of the study design (Figure 2).

In all articles, studies were designed with the assumption that keratometry measurements are the most important factor influencing IOL power calculations in DED, and Yang et al. [13], Hiraoka et al. [20], and Epitropoulos et al. [24] conducted prospective non-randomized studies comparing biometric measurements in DED and non-DED groups. Kim et al. [22] compared DED patients retrospectively in a pretreatment group treated before biometric measurements and a non-pretreatment group not treated before biometric measurements. Rochct et al. [21] prospectively compared outcomes before and after artificial tear drops in the same eye of patients scheduled for cataract surgery. In their subgroup analysis, they evaluated patients with DED with a TBUT of less than 5 s. Hovanesian et al. [23] prospectively compared the results before and after DED treatment of the same eye. All reports concluded that DED affects IOL power calculations.

### 3.1. Preoperative Diagnosis of DED

A summary of the DED diagnoses in each of the studies reviewed in this paper is presented in Table 1. The assessment of subjective symptoms of DED was described in four reports: Yang et al. used the ocular surface disease (OSD) standard patient evaluation of eye dryness (SPEED) II questionnaire [25], Hiraoka et al. used the dry eye-related quality-of-life score (DEQS) questionnaire [26], and Hovanesian et al. used the SPEED questionnaire [27] to score subjective symptoms; whereas Epitropoulos et al. evaluated the subjects by asking them whether they felt their eyes were dry. For objective clinical signs, Epitropoulos et al. used tear osmolarity as the primary objective test item, while TBUT was commonly adopted in the other reports. According to the TFOS DEWS II Definition and Classification Report [6], tear hyperosmolarity is one of the important etiologic causes of DED, but it does not characterize the pathophysiology of DED. Perhaps for this reason, tear osmolarity has not been widely used as the most important test for DED diagnosis in recent reports. However, it is adopted as one of the primary diagnostic tests in the TFOS DEWS II Management and Therapy Report [28]. DED is pathophysiologically classified into two categories, aqueous-deficient dry eye and evaporative dry eye, and the TFOS DEWS II Definition and Classification Report asserts that aqueous-deficient dry eye and evaporative dry eye are not mutually exclusive, so the use of TBUT in the diagnosis of DED is reasonable. Yang et al. measured non-invasive TBUT using an ocular surface comprehensive analyzer (Keratograph 5 M, Oculus, Wetzlar, Germany); Rochct et al., Hiraoka et al., and Hovanesian et al. measured invasive TBUT using slit lamp microscopy. The TBUT values within those reports meet the objective clinical signs criteria of the TFOS DEWS II Diagnostic Methodology Report. Kim et al. did not describe the diagnostic criteria for DED or the instruments used to measure TBUT. Instead, they described the results of TBUT, ocular surface staining score, and Schirmer test value. Judging from those averages, the subjects may have generally met the criteria for objective clinical signs of DED. Epitropoulos et al. used a tear osmolarity of 316 mOsm/L or more as the cut-off for the hyperosmotic group (DED group) and 308 mOsm/L or less as that for the normal group (the non-DED group), on the basis [29] that, for DED diagnosis, 315 mOsm/L is the most specific value and 308 mOsm/L is the most sensitive value. The subjects, in their report, also met the objective clinical signs criteria of the TFOS DEWS II Diagnostic Methodology Report.

### 3.2. Preoperative Management of DED and Refractive Outcomes

A summary of preoperative management of DED and refractive outcomes is presented in Table 2. In three reports, preoperative eye drops were applied to DED patients. Rochct et al. applied an osmotic pressure regulator (artificial tears) containing trehalose and sodium hyaluronate once a minute before biometric measurements. Kim et al. applied 0.5% loteprednol etabonate eye drops four times a day and 0.05% cyclosporine A eye drops twice a day to the pretreatment group for two weeks before biometric measurements. In their study, eyelid scrubbing with warm compression was also performed in the pretreatment group in combination with eye drop therapy. Hovanesian et al. first performed biometric measurements on the patients, then applied lifitegrast 5% twice daily for 28 days prior to performing biometric measurements again. Preoperative management was not performed in the Yang et al., Hiraoka et al., or Epitropoulos et al. studies.

The studies by Yang et al., Hiraoka et al., and Epitropoulos et al. all evaluated the reproducibility of keratometric measurements. In these reports, keratometric values were measured twice before surgery and compared between the DED and non-DED groups. The difference between the first and second measurements in terms of mean keratometric values (an index of predicted spherical equivalent in IOL power calculations) was 0.28 D in the DED group and 0.09 D in the non-DED group as reported by Yang et al. Epitropoulos et al. reported values of 0.28 D for the DED group and 0.13 D for the non-DED group. Hiraoka et al. reported the steep meridian of corneal curvature radius to be 0.21 D for the DED group and 0.14 D for the non-DED group. These results demonstrate that the differences were significantly greater in the DED groups than in the non-DED groups, proving that DED reduces the reproducibility of keratometric measurements. In addition, Yang et al. and Epitropoulos et al. simultaneously showed that the presence or absence of subjective symptoms of DED had no effect on reproducibility. Kim et al. investigated the effect of preoperative DED treatment on postoperative refractive error, which was calculated as the difference between the predicted spherical equivalent derived from IOL power calculations and the measured value at 1 month postoperatively, and found refractive error to be significantly lower in the pretreatment group than in the non-pretreatment group. This was observed in both the Sanders–Retzlaff–Kraff/Theoretical (SRK/T) and Barrett universal II IOL power formulas (0.23 D for SRK/T and 0.24 D for the Barrett universal II in the pretreatment group, and 0.42 D for SRK/T and 0.38 D for the Barrett universal II in the non-pretreatment group). Hovanesian et al. used biometric measurements of the same eye before and after DED treatment to predict the postoperative spherical equivalent. The differences between the preoperative predictions and the actual measurements at 1 month postoperatively were calculated and compared, and the results were found to be significantly lower than predicted due to the posttreatment biometry results. Although specific values for refractive error were not given in this report, the achieved accuracy was 50%, 79%, and 91% in the pretreatment group, and 47%, 71%, and 81% in the non-pretreatment group (within 0.25 D, 0.5 D, and 0.75 D, respectively). The IOL calculation formulas used in this study, however, were not described. Rochct et al. focused on the influence of artificial tears on toric IOL calculation: in the DED group with a TBUT less than 5 s, artificial tear instillation induced a significant change in corneal astigmatism power. The astigmatic error in the IOL calculation was 0.37 D in the artificial tears group and 0.48 D in the no artificial tears group, representing a significant difference between the two groups. This resulted in a change in the optimal cylinder step of the toric IOL in many cases (e.g., T3 to T4), and this was significantly associated with DED. No significant change was found for the astigmatic axis, but cases with an axial change greater than 10 degrees were significantly associated with DED.

## 4. Discussion

For proper DED management, the TFOS DEWS II Definition and Classification Report first recommends a subjective symptom survey using a questionnaire, followed by risk factor assessment, which will help in later DED management, followed by an examination of clinical signs. Then, subtyping and severity assessment of the evaporative-predominant or aqueous-deficient-predominant subtype should be performed to determine where the patient falls on the subtyping spectrum. As a result, an appropriate therapeutic intervention can be determined.

The TFOS DEWS II Diagnostic Methodology Report suggests the five-item dry eye questionnaire (DEQ-5) [30] and the ocular surface disease index (OSDI) questionnaire [31] as the subjective symptom questionnaires necessary for diagnosis. The OSDI was selected for the report as it is currently the most used questionnaire in the world for subjective symptom survey in DED patients, with the validity of the other questionnaires proven through comparison with the OSDI. The DEQ-5, with its simplified content and high discriminative power, was also selected due to its usefulness. Four of the articles reviewed in this study investigated subjective symptoms, and three of them used questionnaires. The questionnaires used in the three articles were the OSD SPEED II questionnaire, the DEQS questionnaire, and the SPEED questionnaire. In other words, none of the six studies conducted the subjective symptom survey recommended in TFOS DEWS II. The TFOS DEWS II Definition and Classification Report [32] on risk factor assessment for DED mentions factors other than female sex and older age. The report states that meibomian gland dysfunction (MGD), hematopoietic stem cell transplantation, computer use, contact lens wear, Sjögren’s syndrome, adverse environmental conditions (pollution, low humidity, etc.), systemic connective diseases, certain medications (antihistamines, antidepressants, anxiolytics, isotretinoin, etc.), and low levels of androgen metabolites have been established as risk factors. No evaluation of these risk factors was performed in the six studies. The clinical signs of DED (objective clinical signs) are defined as the presence of clinical signs when at least one of TBUT, tear osmolarity, or ocular surface staining meets the criteria of TFOS DEWS II. In all six studies, eligible patients met the criteria. According to TFOS DEWS II, the presence of clinical signs without subjective symptoms is classified as either reduced corneal sensitivity or a precursor state to DED, and the possibility that the patients in the six studies may have fallen into these categories cannot be ruled out. However, in contrast to deficiencies in DED diagnosis, improvement in clinical signs was found to improve the reproducibility of keratometric measurements and the accuracy of predicted postoperative spherical equivalent and residual astigmatism values in IOL power calculations. The TFOS DEWS II Definition and Classification Report states that, even when only clinical signs are present, management may be warranted to prevent the development of DED and to optimize the corneal surface, such as before surgery or contact lens wear. In evaluating our findings, in terms of management of DED during IOL calculations, the goal could be to improve clinical signs (especially TBUT) regardless of the presence or absence of symptoms.

Three of the studies (Rochct et al., Kim et al., and Hovanesian et al.) showed management methods that improved clinical signs, and improved accuracy of IOL power calculation was demonstrated in those reports. Nevertheless, there were some problems with the management methods used. Rochct et al. showed that artificial tears 1 min before biometry measurements improved measurement and prediction accuracy in patients with clinical signs, and recommended this method. However, Jensen et al. conducted a similar study in patients scheduled for cataract surgery and reported no significant differences in measurements [33]. Röggla et al. reported low reproducibility of keratometric values at 30 s and 2 min after the instillation of artificial tears and a low reliability of measurements [34]. Therefore, additional research should be undertaken into the use of eye drops 1 min before biometric measurements. Mrukwa et al. reported that corneal astigmatism changes with time after blinking [35], but this was also not taken into account in the report by Rochct et al. Kim et al. applied steroid eye drops and cyclosporine eye drops for 2 weeks before biometric measurements. Although the short application period is an advantage for patients, there is some concern that the need for multiple treatment modalities, such as the use of two types of eye drops and eyelid scrubbing with warm compression, may decrease adherence. Hovanesian et al. used a 4-week application of lifitegrast as a management strategy. Lifitegrast is one of the drugs recommended in Step 2 of the DED management algorithm in the TFOS DEWS II Management and Therapy Report [36]. Lifitegrast acts to reduce inflammation; however, there is a growing consensus that controlling inflammation is not a core strategy for DED treatment, and short-term treatment may not improve the clinical signs in some DED subtypes [37,38]. It is also difficult to assess the short-term management effect of lifitegrast alone as patients were undergoing other DED treatments that were already in place at the time the study began.

Clinical signs of DED (especially TBUT shortening) observed before cataract surgery were found to adversely affect IOL power calculations, but these can be ameliorated by short-term treatment prior to biometric measurements. However, some problems were found with the management methods applied in the selected studies. In this paper, I propose the use of long-acting diquafosol eye drops for 4 weeks as a DED management method for IOL power calculations. The recommended administration of long-acting diquafosol is three times daily, and its efficacy is comparable to that of conventional drugs after four weeks of application [39] with good adherence [40]. Diquafosol is a topical secretagogue included in Step 2 of the management algorithm in the TFOS DEWS II Management and Therapy Report and acts on P2Y_2_ receptors [41]. Receptor activation promotes mucin and water secretion through the Ca^2+^ and Cl^−^ channels [42] and lipid layer thickening [43,44]. This action occurs independently of lacrimal gland function [45]. Diquafosol increases mucin, water, and lipids in the tear fluid, thus stabilizing the tear film. In addition, diquafosol has proliferative and reparative effects on the ocular surface epithelium by increasing the phosphorylation of epidermal growth factor receptors and extracellular signal-regulated kinases [46]. A study reported that diquafosol significantly improved corneal epithelial damage by improving tear secretion and the function of the corneal epithelial barrier [47]. These findings indicate that diquafosol is effective for both aqueous-deficient dry eye and evaporative dry eye. The TFOS DEWS II Definition and Classification Report advocates that “aqueous deficient dry eye and evaporative dry eye exist as a continuum”; therefore, diquafosol is an appropriate agent for use in the short-term preoperative management of DED for IOL power calculations. However, if subtyping and severity assessment results reveal a higher severity of evaporative dry eye due to MGD or aqueous-deficient dry eye due to Sjögren’s syndrome, etc., treatment with diquafosol alone may not be effective in stabilizing the tear film. In such cases, additional treatment such as specialized treatment for MGD (intense pulsed light, vectored thermal pulsation, etc.) or punctal occlusion may be necessary. Further, this preoperative management method has several limitations. First, it has not been validated in clinical trials. Second, it does not consider the intraoperative and postoperative effects of DED on cataract surgical practice. Third, the relationship between the position on the DED subtype classification spectrum and the management efficacy of long-acting diquafosol eye drops remains to be clarified. Additional studies are needed to address these limitations.

One limitation of this review is that the literature search was conducted using a single bibliographic database. However, as PubMed is a reliable database containing the world’s leading medical journals, this was not regarded as a major problem when extracting articles.

## 5. Conclusions

Cataract surgeons must pay more attention to preoperative DED diagnosis and treatment. The study design in the articles in this systematic review should have included assessment of the risk of bias. In addition, based on the TFOS DEWS II, the global standard for DED, the DED diagnoses within each article were inadequate and their preoperative management methods demonstrated some weaknesses. However, improvements in the clinical signs of DED (especially TBUT) were found to improve the accuracy and reproducibility of IOL power calculations. Therefore, as a necessary practice for IOL power determination, an appropriate DED diagnosis should always be made at the time of preoperative screening examination, followed by DED management prior to biometric measurements for patients diagnosed with DED. Subsequent biometric measurements and IOL power calculations would minimize postoperative refractive error in DED patients. Further evidence based on appropriately designed studies is needed for this purpose. In future, many randomized controlled trials should be conducted to establish a standard preoperative management method for DED for IOL power calculations.

## Figures and Tables

**Table 1:** Study design overview.

Author	Type of Study	N	Comparison	Subjective Symptoms of DED	Objective Clinical Signs of DED
Yang et al., 2024 [13]	Non-randomized controlled clinical study	83	DED versus non-DED group	OSD SPEED II questionnaire	TBUT less than 10 s and positive corneal and conjunctival fluorescein staining
Hiraoka et al., 2022 [20]	Observational prospective non-randomized study	114	DED versus non-DED group	DEQS questionnaire	TBUT less than 5 s and positive corneal fluorescein staining
Rochct et al., 2021 [21]	Comparative, monocentric, prospective study	73	Comparison of the same eye before and after eye drops	None stated	Subjects not limited to DED, but 54.8% with TBUT less than 5 s
Kim et al., 2021 [22]	Retrospective observational study	105	Preoperative DED treatment versus no treatment group	None stated	TBUT, ocular surface staining score, Schirmer test (no diagnostic criteria listed)
Hovanesian et al., 2020 [23]	Multicenter, prospective, open-label study	58	Comparison of the same eye before and after DED treatment	SPEED questionnaire	TBUT less than 10 s and positive corneal fluorescein staining
Epitropoulos et al., 2015 [24]	Observational prospective non-randomized study	144	Tear hyperosmolar versus normal osmolarity group	Verbal questions	Hyperosmolarity is tear osmolarity 316 mOsm/L or more, normal is less than 308 mOsm/L

DED: dry eye disease, OSD: ocular surface disease, SPEED: standard patient evaluation of eye dryness, DEQS: dry eye-related quality-of-life score, TBUT: tear film break-up time.

**Table 2:** Preoperative DED treatment, biometry, and research results.

Author	Therapeutic Eye Drops	Length of Treatment	Keratometry Measurement Biometry	Results
Yang et al., 2024 [13]	None	None	OA-2000	Reproducibility of corneal curvature and astigmatic vectors was significantly lower in the DED group.
Hiraoka et al., 2022 [20]	None	None	IOLMaster 500	The measurement repeatability of corneal curvature radius declined in eyes with DED.
Rochct et al., 2021 [21]	Artificial tears	1 min	IOLMaster 700	Predicted astigmatic error improved significantly after eye drops in the group with TBUT less than 5 s.
Kim et al., 2021 [22]	Steroid and cyclosporin *	2 weeks	IOLMaster 500	Preoperative treatment significantly reduced postoperative refractive error and number of refractive surprises.
Hovanesian et al., 2020 [23]	Lifitegrast	4 weeks	IOLMaster 500 or 700	Preoperative treatment significantly increased the accuracy of predicting post-operative spherical equivalent.
Epitropoulos et al., 2015 [24]	None	None	IOLMaster (No model mentioned)	High osmolarity group showed significant variation in mean keratometry values and anterior corneal astigmatism.

DED: dry eye disease, TBUT: tear film break-up time. * Eyelid scrubbing with warm compression was used in addition to eye drop therapy.

## Data Availability

Not applicable.
